# Anti-Mullerian hormone levels decline under hormonal suppression: a prospective analysis in fertile women after delivery

**DOI:** 10.1186/1477-7827-9-98

**Published:** 2011-07-21

**Authors:** Andrea Weghofer, Wolf Dietrich, Iris Ortner, Christian Bieglmayer, David Barad, Norbert Gleicher

**Affiliations:** 1 Department of Obstetrics & Gynecology, Medical University Vienna, Austria; 2 The Center for Human Reproduction (CHR), New York and The Foundation for Reproductive Medicine, Chicago, Illinois, USA; 3 Department of Obstetrics & Gynecology, General Hospital Mistelbach, Austria; 4 General Hospital Moedling, Austria; 5 Department of Medical Laboratory Diagnostics, Medical University Vienna, Austria; 6 Department of Epidemiology and Social Medicine and Department of Gynecology and Women's Health, Albert Einstein College of Medicine, Bronx, New York, USA; 7 Department of Obstetrics, Gynecology and Reproductive Sciences, Yale University School of Medicine, New Haven, Connecticut, USA

## Abstract

**Background:**

AMH's reported stability during periods of hormonal change makes it a practical tool in assessing ovarian reserve. However, AMH declines with age and age-specific cut-offs remain to be established in women with proven fertility. This study aims to determine age-specific ranges of AMH in women with proven fertility.

**Methods:**

Two hundred-ten fertile women, aged 18-40 years, were prospectively recruited for AMH measurements within 14 days after delivery and age stratified into 3 groups (18-30, 31-36 and 37-40 years). Eligibility required spontaneous conception within a maximal period of six months. Autoimmune diseases, chemotherapy, radiation, ovarian surgery and polycystic ovary syndrome precluded inclusion.

**Results:**

95% confidence intervals of AMH declined with advancing female age from 0.9-1.1 to 0.6-0.9 and 0.2-0.4 ng/mL (P < 0.001). AMH levels were not statistically associated with day of blood draw after delivery or pregnancy characteristics. Neither were they predictive of resumption of menses. They, however, at all ages were lower than reported in the literature for infertile patients.

**Conclusions:**

Like infertile populations, fertile women demonstrate declining AMH with advancing age. Uniformly lower levels than in infertile women suggest that AMH levels do not appear as stable under all hormonal influences as previously reported.

## Background

Accurate assessments of ovarian reserve are crucial and allow for appropriate counselling during women's reproductive life spans. Anti-Müllerian hormone (AMH) is increasingly used as diagnostic marker in assessing ovarian reserve (OR). Indeed, we and others reported improved accuracy in predicting oocyte yields and pregnancy potential, compared to baseline follicle stimulating hormone (b-FSH) [1,2].

Anti-Müllerian hormone is produced in granulosa cells of small follicles from primary stage on [3,4]. By inhibiting follicular recruitment [3], AMH exerts regulative functions on folliculogenesis, and serum concentrations are relatively closely reflective of numbers of antral follicles [5,6]. Antral follicle counts are, however, widely believed to be directly proportional to a woman's total follicle pool and, therefore, reflective of her OR [3,5,7]. AMH's reported stability during periods of hormonal change, such as menstrual cycles and in association with pregnancy, supports the assumption that AMH may, indeed, be reflective of a woman's total follicle pool [8-10]. This AMH characteristic also makes it a more practical clinical tool in comparison to other modalities [11].

AMH, however, gradually declines as women age [12,13]. Among infertile patients, substantially higher percentages of women at both extremes of AMH values (i.e., diminished ovarian reserve and polycystic ovary syndrome) are observed. Currently available age-specific normograms were usually established in infertile patient populations [14]. In an attempt to establish age-specific values in a fertile cohort, Shebl et al. retrospectively investigated AMH levels in women undergoing IVF due to male factor or idiopathic infertility. Their results, however, clearly demonstrate compromised AMH levels (i.e. ≤1 ng/ml) in 5% of women throughout all ages. They, therefore, conclude that even young 'presumbly healthy' women undergoing IVF are at risk of diminishing ovarian reserve. Moreover, no data on pregnancy potential in those women are given [14]. Age-specific AMH cut-offs, established in a population with proven fertility, would, therefore, be useful to accurately diagnose normal age-specific ovarian reserve or prematurely declining ovarian reserve, respectively. To determine such age-specific cut-offs, AMH measurements were initiated in a cohort of women shortly after delivery after spontaneous conception within six months of unprotected intercourse.

## Methods

Between April 2008 and September 2009, prospective recruitment of 210 women with proven fertility in three age strata (n = 70 in each group; Group 1, 18-30 years; Group 2, 31-36 years; Group 3, 37-40 years) was performed. Recruitment occurred within four and consecutive blood draw for AMH measurement within 14 days after delivery at the Department of Obstetrics & Gynecology, Medical University Vienna, Austria. Follow up telephone interviews were completed six to 24 months after delivery.

Women were eligible for enrollment if they had conceived spontaneously within six months and were excluded with a history of infertility, autoimmune disease, chemo/radiation therapy, ovarian surgery and polycystic ovary syndrome. All AMH measurements were performed at the Department of Medical Laboratory Diagnostics, Medical University Vienna, using the DSL Active MIS/AMH assay (Beckman Coulter Inc., USA).

After the completion of recruitment, one-hundred-and-four women were available for follow up telephone interview six to 24 months after delivery and asked about current contraceptive methods utilized, duration of breast feeding and resumption of menses.

In order to establish required sample sizes, differences in age-related AMH levels were calculated according to Shebl et al. [14]. To detect a comparable decline of AMH between the Groups with 80% power and 0.1% level of significance, a sample size of 62 per group was needed. We, therefore, decided to recruit 70 women per age strata.

Baseline characteristics of patients and AMH levels were compared between the Groups 1 to 3, using analyses of variance or chi square, as appropriate. Variables that were not normally distributed were first log transformed, and are reported as geometric means and 95% confidence intervals (CI). Normally distributed variables were reported as means ± standard deviation (SD). We used linear regression to assess effects of baseline characteristics, postpartum AMH on pregnancy and postpartum factors. A p-value < 0.05 was considered statistically significant. Statistical analyses were performed using SPSS version 17.0.

Institutional Review Board (IRB) approval was obtained from the Ethics Committee at the Medical University Vienna. After recruitment, all patients signed appropriate informed consents.

## Results

Baseline characteristics in the three age strata are shown in Table 1. Mean age for all study patients was 32.4 ± 5.7 years. Mean postpartum AMH levels were 0.6 ng/ml (95% CI, 0.5-0.7). AMH determinations were made at an average of 3.4 ± 2.1 days after delivery. Patients delivered at a mean gestational age of 38.0 ± 3.2 weeks.

**Table 1:** Baseline characteristics & outcome data of 210 fertile women within 14 days after delivery according to age

(n = 70 per group)	Group 1		Group 2		Group 3		P
	(18-30 years)		(31-36 years)		(37-40 years)		
**Female age (years)**	25.7 ± 3.5	[24.8-26.5]	33.4 ± 1.6	[33.0-33.8]	38.2 ± 1.1	[38.0-38.5]	<0.01
**Postpartum AMH (ng/ml)**	0.9	[0.7-1.1]	0.7	[0.6-0.9]	0.3	[0.2-0.4]	<0.001
**Delivery to AMH draw (days)**	3.4 ± 2.3	[2.8-3.9]	3.6 ± 2.3	[3.1-4.2]	3.1 ± 1.6	[2.8-3.5]	n.s.
**Delivery to resumption of menses (months)**	3.4	[2.6-4.4]	4.5	[3.7-5.3]	5.3	[3.9-6.8]	<0.05
**Gestational age at delivery**	37.9 ± 3.2	[37.2-38.7]	38.0 ± 2.9	[37.3-38.7]	38.1 ± 3.6	[37.2-38.9]	n.s.

*Normally distributed variables are presented as mean ± SD and 95%CI, not normally distributed variables are presented as log transformed means with 95 CI*.

AMH levels were negatively associated with age (p < 0.0001). Average AMH levels by age group were: 0.9 ng/ml (95% CI, 0.7-1.1) in Group 1, 0.7 ng/ml (95% CI, 0.6-0.9) in Group 2, and 0.2 ng/ml in Group 3 (95% CI, 0.2-0.4). There was no difference between age groups in gestational age at delivery, mode of delivery, fetal gender, birth weight and intervals between deliveries and AMH draw.

Due to the considerably long time period of up to 24 months between recruitment and follow up, only 104 women were available for a telephone interview. Among those, menses resumed after mean duration of 3.4 months (95% CI, 2.6-4.4) in Group 1, after 4.5 months (95% CI, 3.7-5.3) in Group 2, and after 5.3 months (95% CI, 3.9-6.8) in Group 3. The interval to onset of menses increased significantly with increasing age (p < 0.03) and was proportionally delayed with the duration of breast feeding (p < 0.001). Women who completed follow up were evenly distributed between the age strata.

Using linear regression we found no significant association between AMH levels and onset of menses. After constructing multiple linear regression models, adjusted for age, duration of breast feeding and hormonal contraception usage, there was still no significant effect of AMH levels on onset of menses.

## Discussion

Our results demonstrate gradually declining AMH levels with age in fertile women, similar to declines reported in infertile populations [15]. They, however, also show substantially lower AMH levels postpartum than reported in infertile patients [10,14]. This, most likely, reflects a suppressive effect of pregnancy/puerperium on growing follicle cohorts and, thus, AMH. Though patient age and duration of breast feedings significantly influenced intervals to resumption of menses, AMH levels did not.

This study investigated only women with proven fertility. It, therefore, appears unlikely that the observed, apparently suppressed, AMH levels postpartum reflect their actual total follicle pool. The substantially reduced AMH concentrations postpartum may, therefore, mirror lower folliculogenesis after delivery. These assumptions are supported by the delay of menopause in multiparous women [16,17].

Recent findings of Nelson et al. are in accordance with the assumption of a suppressive effect of hormonal changes on AMH levels: They reported declining AMH levels during second and third trimesters of pregnancy [18], contradicting prior observations that claimed AMH to be stable during pregnancy and puerperium [19,20]. Also contrary to prior reports [21], AMH may also be suppressed with oral contraceptives [22]. Van den Berg et al. recently reported that AMH on day seven of contraception free intervals does not correspond to AMH values in subsequent natural cycles. They reported not only increasing AMH levels immediately after cessation of contraception, but even higher levels after two months from cessation [22].

Data from women with polycystic ovary syndrome (PCOS) after ovarian drilling further support the point: Shebl et al. report decreased AMH levels shortly after ovarian drilling, when compared to pre-surgery levels. AMH, however, returned to approximately original levels within six months from surgery [23].

All of these data raise questions as to what extent, and under which conditions, AMH is reflective of the total follicle pool. It, indeed, appears possible that AMH primarily reflects growing follicles (i.e. an actively maturing follicle *cohort*), but not - or not under all circumstances - the complete follicle *pool*. Further studies are, therefore, warranted to evaluate factors that might possibly interfere with AMH secretion or rather under which conditions AMH concentrations can be utilized to assess ovarian reserve.

This study, as one would expect, also demonstrates a significant decline of AMH levels with age in fertile women. Fertile women, further, show a significant correlation between age and resumption of menses - the older a woman is at the time of delivery the longer it takes to resume menses. Latter correlation could reflect declines in ovarian reserve with advancing female age, and one, therefore, would expect a comparable association between postpartum AMH levels and resumption of menses (corrected for breast feeding and hormonal contraceptive usage), if AMH, indeed, was reflective of total ovarian follicle pools. Our data, however, clearly failed to demonstrate such an association. Though data on resumption of menses were not available for the whole cohort of 210 women, this lack of association was seen in all three age groups.

## Conclusions

Like infertile populations, fertile women demonstrate declining AMH with advancing age. Uniformly lower levels than in infertile women suggest that AMH levels do not appear as stable under all hormonal influences as previously reported.

## Competing interests

The authors declare that they have no competing interests.

## Authors' contributions

AW, WD Substantial contributions to conception and design, acquisition, analysis and interpretation of data, drafting the article, final approval of the version to be published. IO, CB Substantial contributions to acquisition and contributions to analysis of data, revising the article critically for important intellectual content, final approval of the version to be published. DHB Substantial contributions to conception of the study, analysis of data, revising the manuscript critically for important intellectual content, final approval of the version to be published. NG Substantial contributions to conception and design, analysis and interpretation of data, revising the manuscript critically for important intellectual content, and final approval of the version to be published. All authors read and approved the final manuscript.
